# Bleeding resuscitation in the ambulance service, an observational study of standard care in Sweden

**DOI:** 10.1186/s13049-025-01439-7

**Published:** 2025-07-25

**Authors:** G. Skallsjö, G. Sandström, JG. Sato Folatre, AM. Harstad, A. Åström Victorén, E. Sömoen, E. Höglund, J. Haglund, J. Sköld, T. Bergström, C. Magnusson, K. Wibring, B. Romlin, A. Wikman

**Affiliations:** 1 Helicopter Emergency Medical Service, Västra Götalandsregionen, Gothenburg, Sweden; 2 Department of Anaesthesiology and Intensive Care, Institute of Clinical Sciences, Sahlgrenska Academy, University of Gothenburg, Gothenburg, Sweden; 3 Department of War Studies, Swedish Defense University, Stockholm, Sweden; 4 Department of Emergency Medical Service, Region Jönköping, Jönköping, Sweden; 5 Department of Anaesthesiology and Intensive Care, Central Hospital of Karlstad, Karlstad, Sweden; 6 Department of Ambulance Care, Faculty of Medicine and Health, Örebro University, Örebro, Sweden; 7 Department of Ambulance and Prehospital Care, Region Halland, Sweden; 8 Institute of Health and Care Sciences, Sahlgrenska Academy, University of Gothenburg, Gothenburg, Sweden; 9 Department of Molecular and Clinical Medicine, Gothenburg University, Gothenburg, Sweden; 10 Department of Prehospital Emergency Care, Sahlgrenska University Hospital, Gothenburg, Sweden; 11 Helicopter Emergency Medical Service, Region Norrbotten, Gällivare, Sweden; 12 Department of Anaesthesiology, Gällivare Hospital, Gällivare, Sweden; 13 Department of Paediatric Anesthesia and Intensive Care, Sahlgrenska Academy, The Queen Silvia Children’s Hospital, University of Gothenburg, Gothenburg, Sweden; 14 Department of Emergency Medical Service, Region Norrbotten, Luleå, Sweden; 15 Department of Emergency Medical Service, Västra Götalandsregionen, Trollhättan, Sweden; 16 Clinical Immunology and Transfusion Medicine, Department of Medicine Huddinge, Karolinska University Hospital, Karolinska Institutet, Stockholm, Sweden; 17 Helicopter Emergency Medical Service, Västra Götalandsregionen, Kungälvs Sjukhus, Lasarettsgatan 1, Kungälv, 442 83 Sweden

**Keywords:** Ambulance service, Prehospital fluid resuscitation, Prehospital coagulopathy, Shock index, Transfusion with blood products, Trauma induced coagulopathy

## Abstract

**Background:**

The ambulance service in Sweden has most often only crystalloids as resuscitation, even though restrictive use of clear fluids in bleeding patients is recommended. The aim of this study was to describe the treatment and outcome of bleeding patients treated by the ambulance service.

**Methods:**

This was a prospective observational multi-center study. Ambulance organizations in six different regions were invited to participate, each of them for a period of six months. Adult bleeding patients where fluid resuscitation with crystalloids was initiated by the ambulance service was consecutively included. Prehospital data on type of bleeding, mechanism and severity of injury, vital signs, estimated bleeding volume, treatment and transport time was collected from the ambulance service. Results from laboratory tests and data of transfusion requirements and mortality were obtained from the medical records, after hospital admission.

**Results:**

181 patients were resuscitated with crystalloid fluids by the ambulance service and were included in the study. Gastrointestinal bleeding was the cause of fluid resuscitation in 48% of the patients and bleeding due to trauma in 23%. A high proportion of the patients (41%) had a coagulopathy upon admission at the hospital, defined as prothrombin time > 1,2, platelet count < 150 × 10^9^/L and/or activated prothrombin time > 32 s. Shock Index (SI) was 1.2 (mean) (SD 0.4). The mean volume of crystalloid fluids administered was 626 mL (SD 366), with one third of the patients receiving 1000 mL or more. Tranexamic acid was administered to 28% of the patients. Blood transfusions were given to 50% of the patients upon hospital admission. SI more than 1.3 predicted need of blood transfusions and bleeding > 500 mL predicted increased 24 h mortality. The overall 24-hour mortality was 7.2% and in patients with blood loss greater than 500 mL, the mortality rate was 12.1%.

**Conclusion:**

In this study gastrointestinal bleeding and trauma were the leading causes of severe prehospital bleeding. Blood loss over 500 mL and Shock Index above 1.3 were key predictors of poor outcome, highlighting the potential benefit of earlier blood product administration.

**Trial registration:**

Clinical trial number: Not applicable.

## Introduction

The prehospital management of bleeding patients is challenging. Early resuscitation with blood products has been shown to improve survival in severe bleeding cases [1–4], yet their availability in ambulances is limited due to short storage times and logistical constraints. Instead, the general ambulance services primarily rely on crystalloids, even though the recommendation is restrictive use of crystalloids in bleeding patients, since it may impair coagulation and worsen the prognosis [5, 6]. Fluid boluses is used to mitigate the effect of hypovolemic shock by increasing organ perfusion, but with risk of worsening coagulopathy in a bleeding patient [7, 8]. 

An alternative to fresh blood products is freeze- or spray-dried plasma, which can be stored for up to two years at ambient temperature. However, studies on its effectiveness have yielded conflicting results [9], with some studies showing reduced mortality with prehospital blood products, but rePHILL, a RCT, finding no significant benefit between administration of red blood cells and freeze dried plasma compared to saline [10]. In a previous randomized study, 9,8% reduced mortality was shown with resuscitation with plasma. The patients treated with plasma had also a lower prothrombin time (PT-INR) and lactate than the group receiving crystalloids [11, 12]. It is suggested that plasma may be beneficial in long transport time [13]. The variability in study design, patient population, and transport time makes it difficult to determine when and which blood products should be used prehospitally.

Approximately 4.5 million people are estimated to die each year from injuries [14]. Bleeding is considered to cause 35% of all prehospital deaths occurring among injured patients. Only severe central nervous system injuries have a higher mortality rate with 45% of all deaths occurring before the patient reaches the hospital [15].

Acute traumatic coagulopathy can be seen in at least 25% of severely injured patients who are admitted to a trauma center [16–18]. This early coagulopathy can be aggravated by extensive use of crystalloid fluids. Dilution is often considered a likely cause [18] although the exact mechanisms and level of aggravation are largely unknown. Early administration of Tranexamic Acid (TXA) has shown to decrease mortality among bleeding patients [19].

Most research on prehospital haemorrhage management focuses on trauma, but other causes, such as gastrointestinal bleeding and obstetric bleeding can also be severe, particularly in regions with long transport times. Many factors influence survival, including response time, injury mechanism, comorbidities and resuscitation strategies.

In Sweden with a population of approximately 10 million inhabitants there are approximately 300 ambulance stations based in different regions. The treatment regimens for bleeding patients differ slightly, regarding indications on when to start fluid resuscitation, but all protocols include crystalloids as resuscitation fluid and administration of tranexamic acid for patients with severe bleeding.

This study aimed to describe standard prehospital resuscitation practices in Sweden and evaluate patient outcomes, including mortality, severity of coagulopathy, blood transfusion requirements within 24 h, and potential predictors of poor outcome.

## Methods

### Study design

This is a prospective observational multicenter study of standard care in prehospital bleeding. Ambulance organizations in different Swedish regions, were invited to participate, representing both rural and densely populated regions. In all, 6 regions with a total of 100 ambulance stations were able to participate in the study. The participating ambulance services were staffed with ambulance nurses.

Patients that had a bleeding requiring fluid resuscitation where eligible for enrolment in the study. Standard operating procedures of the ambulance service for fluid resuscitation were applied. This means that no separate intervention or adjustment of normal routines was performed.

### Inclusion of patients

Adult (≥ 18 years) bleeding patients requiring fluid resuscitation were consecutively included over six months, starting from the first patient included in each of the participating ambulance service organizations, during the period September 2022 to June 2023. The start of the inclusion was not entirely synchronized among the participating organizations. This was due to our own limitations in providing the necessary information and training to the ambulance organizations. Some ambulance organizations required more time to prepare for inclusion, therefore there were some delays in various regions.

The ambulance service fluid resuscitation protocols differ slightly between health care regions. In general, there is quite a threshold before fluid resuscitation starts. The “hypotensive resuscitation regime” taken from Damage Control Resuscitation (DCR) is commonly used and the tradition in the ambulance service is nowadays a restrictive use of crystalloid fluids. Most ambulance care providers use a SBT < 90 mmHg in combination with a reasonable mechanism of injury and/or visible blood loss to trigger the fluid resuscitation program.

### Study protocol

All included patients were registered in a case report form (CRF) by the ambulance crew at the end of the mission/assignment. The CRF included demographic data, mechanism of injury, vital signs (highest and lowest pulse and blood pressure), temperature, estimated blood loss, volume and type of fluids administered, if TXA was administered and time from arrival at the scene until the patient was handed over at the hospital. The severity of the injuries was assessed in accordance with National Advisory Committee for Aeronautics (NACA) on a scale of 0 to 7, the minimum being 0 (unharmed) and the maximum being 7 (deceased). NACA score of 4 and above indicates a possible life-threatening injury or medical condition. Shock Index (SI) was calculated by dividing heart rate with systolic blood pressure.

The second part of the CRF was registered by a local trial investigator and included transfusion of blood products during the first 24 h, laboratory tests upon arrival at the hospital together with 6- and 24-hour mortality.

Coagulopathy was defined as prothrombin time > 1,2, platelet count < 150 × 10^9^/L and/or activated prothrombin time > 32 s [20–22].

The CRFs were then deidentified, encrypted and included in a high security research database. The data were then exported for statistical analysis.

### Statistical methods

Descriptive statistics are presented as frequencies (n, %) for categorical variables and as means with standard deviations (Mean ± SD) or medians with interquartile ranges (Median [Q1; Q3]) for continuous variables, depending on the data distribution.

To assess the relationships between variables, Spearman’s rank correlation coefficient (rs) was employed for all unadjusted correlations, given its non-parametric nature and suitability for ordinal data or data that do not meet the assumptions of normality. For adjusted correlations, Spearman’s partial correlation coefficient was utilized, controlling for the confounding effect of age.

To identify predictors of dichotomous outcomes, both univariable and multivariable logistic regression analyses were performed. Results are presented as Odds Ratios (OR) with corresponding 95% Confidence Intervals (CI), and the performance of the models was evaluated using the area under the Receiver Operating Characteristic (ROC) curve (AUC), based on the raw data rather than stratified categories.

The Odds Ratio (OR) represents the ratio of the odds with a one-unit increase in the predictor variable. Statistical significance was determined at a 5% level, and all tests were two-sided. Data analyses were carried out using SAS Version 9.4 (SAS Institute, Cary, NC, USA).

### Human ethics and consent to participate

The study was conducted in accordance with the World Medical Association, Declaration of Helsinki. Ethical approval for the study was given by the Swedish Ethical Review Authority (Dnr 2021 03156). Informed consent from the patients was not required since no active intervention was undertaken apart from the Standard Operating Procedures of the ambulance service.

## Results

### Patient demographics and causes of bleeding

**Fig. 1:**
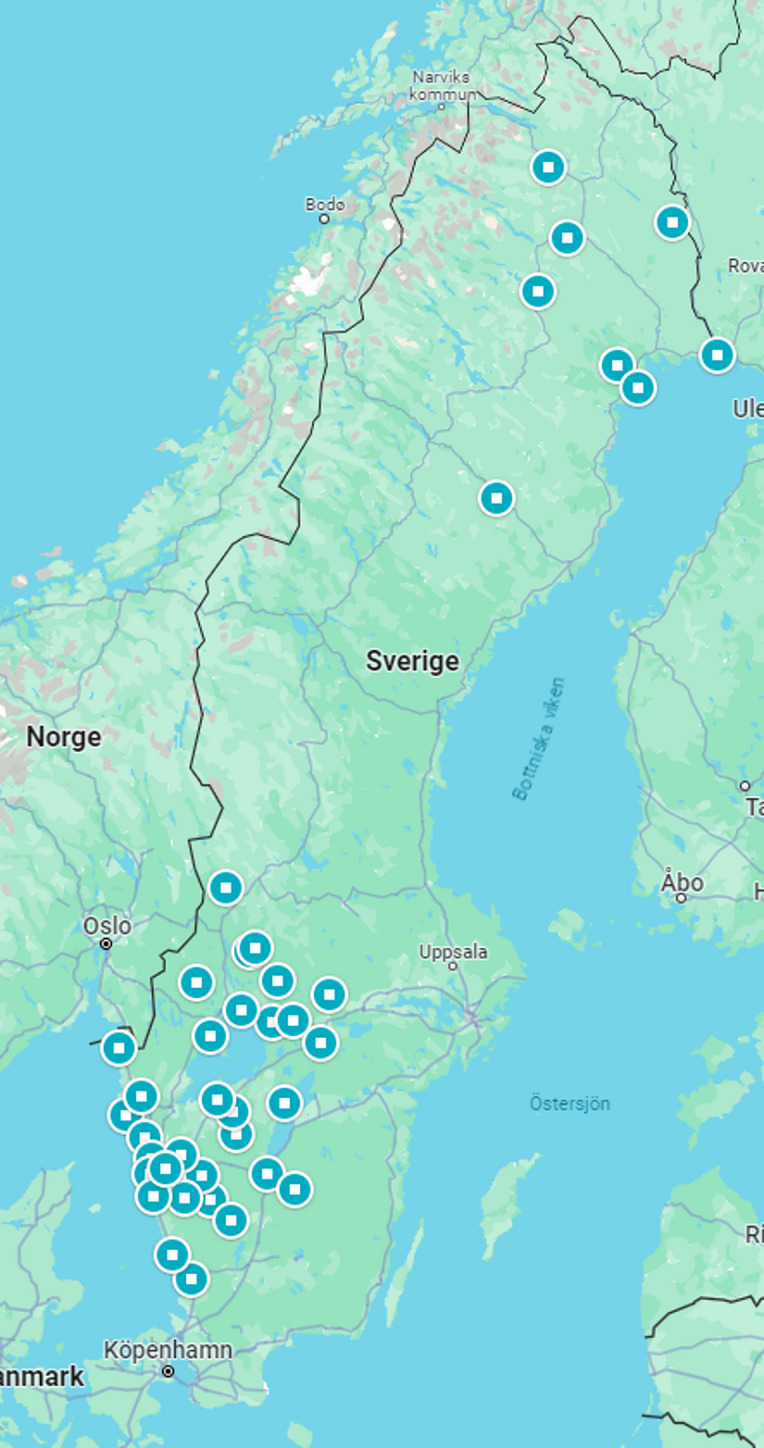
Geographical distribution of the participating 100 ambulance stations in 40 different locations in six regions in Sweden

A total of 181 patients from 40 different geographical locations were included in the study, covering both rural and urban areas, presented in Fig. 1. The mean age of the patients was 65.5 (SD 22.5) years, and 58% were male.

The most common cause of bleeding was gastrointestinal bleeding, which accounted for 87 patients (48.1%). Trauma, including both blunt force and penetrating injuries, was responsible for 42 patients (23.2%), while gynaecological or obstetric bleeding contributed to 25 patients (13.8%). Other causes, such as severe ENT-bleeding, urinary tract bleeding, and postoperative abdominal haemorrhage, made up of 24 patients (13.4%), with aortic aneurysms being the least common with 3 patients (1.7%), as presented in Table 1.

**Table 1:** Patient characteristics

Included patients – no. (%)	181 (100%)
Age - mean (SD)	65.5(22.5)
Gender – no. (%)	
Female	76 (42.0%)
Male	105 (58.0%)
Mechanism of injury or cause of bleeding – no. (%)	
Blunt trauma	25(13.8%)
Penetrating trauma	17(9.4%)
GI-bleeding	87(48.1%)
Gyn/Obstetric	25(13.8%)
Aortic aneurysm	3(1.7%)
Other cause	24(13.4%)
Severity of bleeding – no. (%)	
< 500 mL	82(45.6%)
> 500 mL	99(54.4%)
Tranexamic acid (TXA) – no. (%)	
Yes	51(28.2%)
No	128(70.7%)
Unknown	2
Systolic blood pressure (mmHg) - mean (SD)	
Highest	112 (27.8)
Lowest	86 (24.3)
Heart rate - mean (SD)	
Highest	96 (28.6)
Lowest	81 (24.8)
Shock Index - mean (SD)	1.2 (0.41)
Temperature (°C) - mean (SD)	35.7 (5.2)
Ringer Acetate – no. (%)	181 (100%)
Volume (ml) - mean (SD)	623 (360.6)
Saline – no. (%)	2 (4.5%)
Volume (mL) - mean (SD)	65 (49.2)

### Blood loss and hemodynamic parameters

According to the study-protocol the volume of the bleeding was estimated to be above or below 500 mL, 99 patients (54.4%) had an estimated bleeding of more than 500 mL. Gastrointestinal bleeding was the leading cause in those with a bleeding in excess of 500 mL, with 46 (46.4%) of the patients. Among the trauma patients 31 of 42 (73.8%) had a bleeding of more than 500 mL which is a high proportion compared to other causes of bleeding. In patients bleeding more than 500 mL, a lower SBT was observed, with a mean value of 80 mmHg (SD 25.3) vs. 90 mmHg (SD 22.6) among the patients with a bleeding less than 500 mL. The mean temperature was also slightly lower 34.9 C° (SD 7.1) vs. 36.7 C° (SD 0.8).

**Fig. 2:**
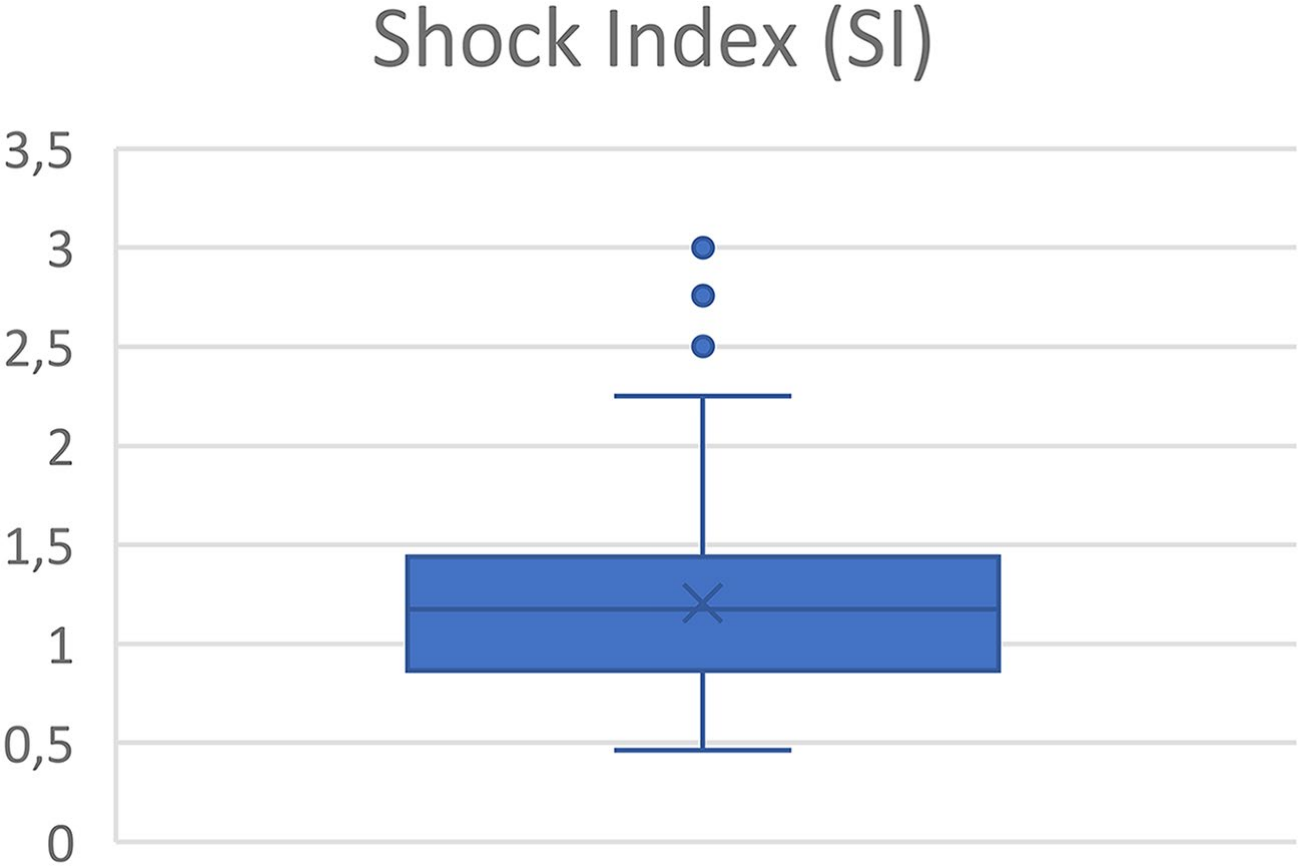
Shock Index (SI) in the patient cohort was 1.2 *±* 0.4 (mean *±* SD), indicating a moderate to high degree of circulatory impairment. 32.6% (*n* = 59) of the patients had SI 1.3 or higher

The mean Shock Index in the total patient cohort was 1.2 (SD 0.4), indicating a moderate to high degree of circulatory impairment. A total of 32.6% of patients had a SI of 1.3 or higher, suggesting an increased risk of exsanguination. SI is presented in Fig. 2.

### Prehospital treatment and fluid resuscitation

The average volume of crystalloid fluid administered was 623 mL (SD 366.5) with 72 patients (35.6%) receiving at least 1000 mL. Tranexamic acid (TXA) was administered to 51 patients (28.3%), while the remaining 128 (71.5%) did not receive it. When we divide the material according to the origin of bleeding, 87 patients were found with GI-bleeding. Of those 87 patients, 21 (24%) received TXA. Of the remaining 94 patients with bleeding of other causes, 30 (32%) received TXA.

**Fig. 3:**
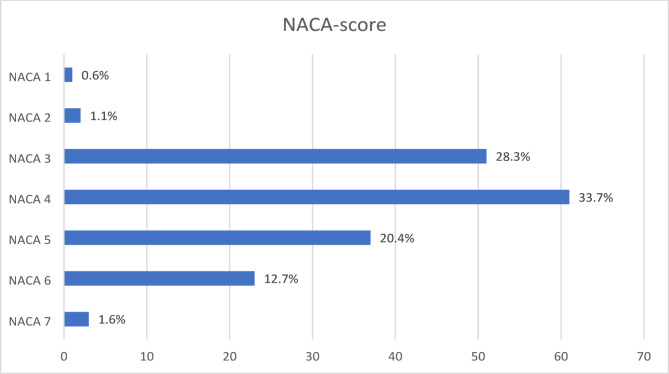
Distribution of National Advisory Committee for Aeronautics (NACA) score, shown as number of patients and percent in each category, where a score of ≥ 4 indicates a potentially life-threatening condition

The severity of the patients’ conditions was reflected in their NACA scores, with a mean of 4.19. A total of 124 patients (68.5%) had a NACA score of 4 or higher, indicating potentially life-threatening injuries or conditions. Presented in Fig. 3. The mean prehospital time, including time spent at the scene and transport to the hospital, was 53 min (SD 27.1), with a median of 49 min and a range of 9 to 180 min.

### Laboratory findings and hospital treatment

All laboratory findings are shown in Table 2. The mean hemoglobin value 102.4 (SD 28.0) confirms anaemia, the elevated PT-INR 1.48 (SD 1.16) impaired coagulation and an elevated lactate value 3.84 (SD 3.5) indicates a hypoperfusion condition at arrival at the hospital.

The overall 24 h mortality was 7.2% and in the group with bleeding above 500 mL 12.1%.

Coagulopathy was detected in 41.4% of all patients and is presented in Table 3.

**Table 2:** Laboratory findings at hospital arrival

	No of patients	Mean (SD)	Reference values	
Hemoglobin (g/L)	176	102.4 (28.0)	134–170	
EVF (L/L)	92	0.32 (0.1)	0.39–0.5	
Platelet count (x 10^9^/L)	156	240 (103.5)	150–348	
PT-INR (ratio)	131	1.48 (1.2)	0-1.2	
aPTT (s)	123	34 (15.5)	24–32	
Lactate (mmol/L)	124	3.84 (3.5)	0.5–1.6	
ASAT (µkat/L)	67	1.0 (1.4)	0.25–0.75	
ALAT (µkat/L)	119	0.7 (1.0)	0.15–1.1	
Bilirubin (µmol/L)	117	16.8 (52.0)	5–25	
pO2 (kPa)	57	11.3 (10.6)	10–14	
pCO2 (kPa)	122	5.7 (1.21)	4.7-6.0	
pH	133	7.33 (0.1)	7.35–7.45	
Sodium (mmol/L)	158	137 (4.3)	137–145	
Potassium (mmol/L)	153	4.3 (0.8)	3.6–4.6	
Creatinine (µmol/L)	155	134 (142.2)	60–105	
Fibrinogen (g/L)	17	2.9 (1.1)	2.0–4.0	

**Table 3:** Endpoints, no of patients (%) (*N* = 181)

Mortality overall 24 h	13 (7.2%)
Mortality 24 h > 500 mL bleeding *	12 (12.1%)
Shock Index > 1.3	59 (32.6%)
Coagulopathy overall	75 (41.4%)
PT-INR > 1.2	14 (7.7%)
Platelet count < 150 × 10^9^/L	9 (5.0%)
aPTT > 32 s	16 (8.8%)
PT-INR > 1.2 and Platelet count < 150 × 10^9^/L	4 (2.2%)
PT-INR > 1.2 and aPTT > 32 s	19 (10.5%)
Platelet count < 150 × 10^9^/L and aPTT > 32 s	3 (1.7%)
PT-INR > 1.2 and Platelet count < 150 × 10^9^/L and aPTT > 32 s	10 (5.5%)

*Data is presented for 99 patients who all had a bleeding more than 500 mL

In Table 4, blood products administered in 24 h at hospital are presented separated into red blood cells (RBCs), plasma and platelets. 92 patients were transfused with 3.76 (mean) (SD 4.2) units of RBCs, (median 3, range 1–34), 28 subjects received 5.18 (mean) (SD 8.5) units of plasma, (median 3, range 1–45) and 11 subjects received 2.27 (mean) (SD 2.0) units of platelets, (median 2, range 1–8).

**Table 4:** Patients treated with blood transfusions in the first 24 h (*N* = 92)

	No of patients	No of units mean (SD)	No of units median (range) IQR (Q1;Q3)
RBC	92	3.76 (4.2)	3 (1–34) (2–4)
Plasma	28	5.18 (8.5)	3 (1–45) (1-5.25)
Platelets	11	2.27 (2.0)	2 (1–8) (1–2)

### Correlations and predictive factors

Statistically significant correlation was found between estimated bleeding > 500 mL and SI (r_s_= 0.17, *p* < 0.05), estimated bleeding > 500 mL and need for transfusion within 24 h (r_s_= 0.24, *p* < 0.005) and SI and need for transfusion within 24 h (r_s_= 0.22, *p* < 0.05). ), as well as estimated bleeding > 500 mL and TXA given (r_s_ = 0.29, *p* < 0.005). Correlations to outcome variables was not shown neither for transport time nor for treatment with crystalloids or TXA.

All variables were adjusted for age. The correlation coefficients are presented in a correlation matrix in Table 5.

**Table 5:** Correlation matrix

	Mortality 24 h	Coagulopathy	SI	Transfusion 24 h	TXA given
Time arrival at hospital	-0.04 0.595	-0.05 0.543	0.057 0.478	0.04 0.620	-0.079 0.302
Volume crystalliod fluid	0.05 0.530	-0.05 0.530	0.10 0.206	0.012 0.878	-0.23 0.002**
Estimated bleeding > 500 mL	0.17 0.019*	0.001 1.000	0.17 0.030*	0.24 0.001**	0.29 0.0001**
TXA given	0.01 0.891	0.086 0.330	0.11 0.188	0.04 0.556	xx
Shock Index (SI)	0.12 0.134	0.033 0.722	xx	0.22 0.006*	0.11 0.188

*p < 0.05, **p < 0.005, Spearmans Correlation Predictors against outcome variables. Adjusted for age

Correlation Coefficients followed by *p*-value

**Fig. 4:**
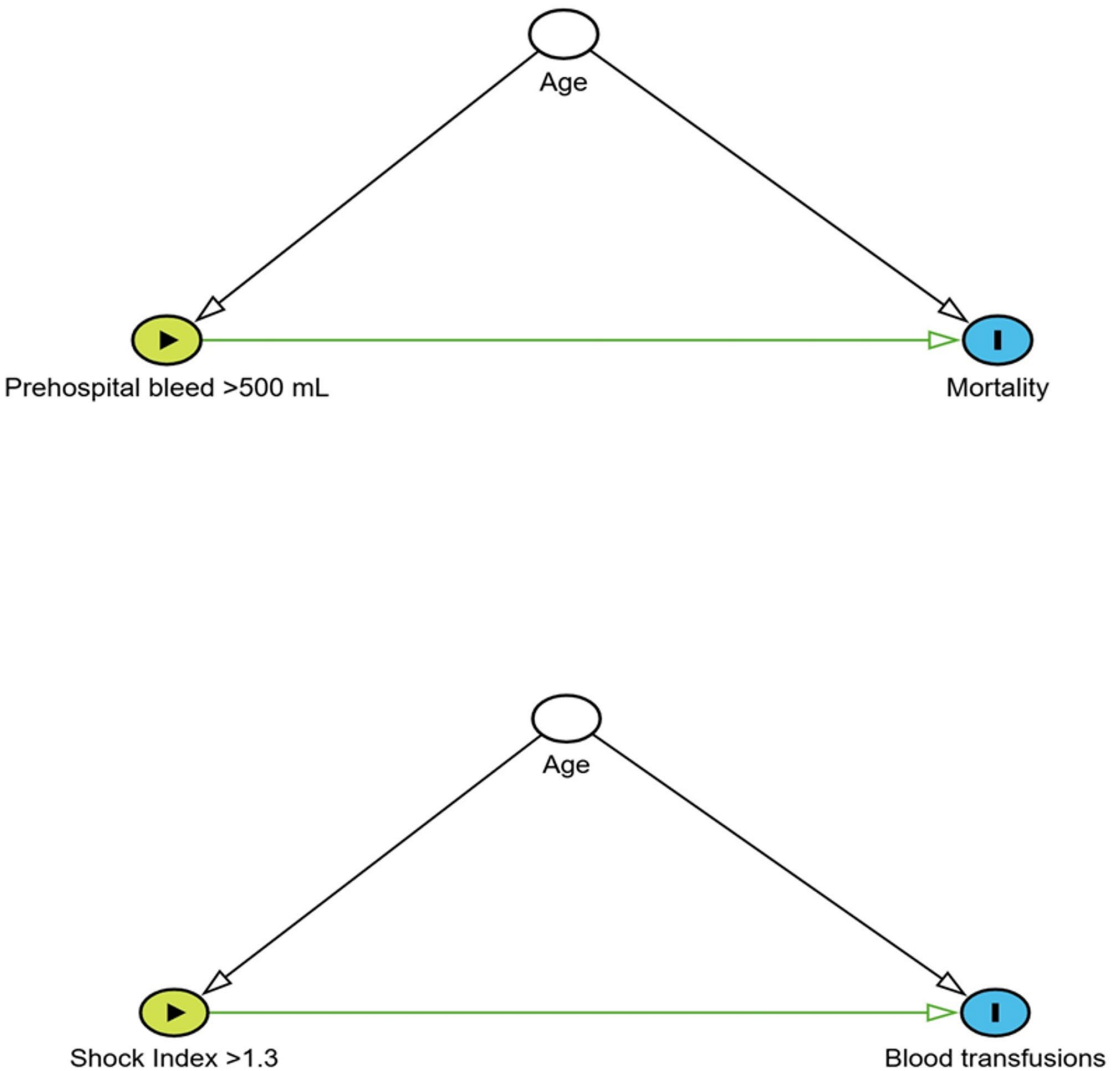
Examples of simple directed acyclic graphs (DAGs) to visualize causality and confounders. Blood loss exceeding 500 mL, adjusted for age, was a predictor of 24-hour mortality (OR 8.28, 95% CI 1.03–66.8, *p* = 0.047) (**4a**) and Shock index (SI) > 1.3, adjusted for age, was a predictor for blood transfusion within 24 h (OR 3.04, 95% CI 1.31–7.08, *p* = 0.0097) (**4b**)

Bleeding > 500 mL, adjusted for age, is a predictor for mortality in 24 h OR 8.28 (95% CI 1.03–66.8, *p* = 0.047). SI > 1.3, adjusted for age, is a predictor for blood transfusions in 24 h OR 3.04 95% CI 1.31–7.08, *p* = 0.0097, Fig. 4a and b.

## Discussion

This study highlights gastrointestinal bleeding and trauma as the leading causes of severe bleeding. A high proportion of patients presented with coagulopathy and an elevated Shock Index, yet only a minority received tranexamic acid. The 24-hour mortality rate was 7.2%, increasing to 12.1% for those with blood loss exceeding 500 mL. Significant predictors of mortality and the need for blood transfusion included blood loss over 500 mL and a Shock Index of 1.3. However, coagulopathy, TXA administration, and transport time did not significantly impact these outcomes.

More than 40% of patients had impaired coagulation upon hospital arrival, a higher rate than typically reported in trauma patients [16–18]. This difference may stem from the inclusion of older patients with underlying conditions requiring anticoagulants. Patients received an average of 600 mL of crystalloids, which could have contributed to coagulopathy in already hemodynamically compromised individuals.

Anaemia was common, with a mean haemoglobin level of 102.4 g/L. While 50% of patients received red blood cell transfusions, only 16% received plasma and 6% received platelets, deviating from balanced transfusion guidelines. This discrepancy may be due to logistical challenges, as plasma and platelets require more preparation time compared to readily available red blood cells. Additionally, some patients may have been stabilized before receiving additional components.

Shock Index is a key predictor of transfusion needs and mortality, with a normal value of 0.7 or lower [23]. In this study, the mean Shock Index was 1.2, with 32.6% of patients exceeding 1.3, indicating a moderate to high degree of circulatory instability. These findings may support that more aggressive prehospital fluid resuscitation preferably with blood products could be beneficial.

Elevated lactate levels (mean 3.84 mmol/L) indicated hypoperfusion, and mean body temperature was slightly reduced at 35.7 °C, with even lower values (34.9 °C) in those with severe bleeding. Hypothermia can exacerbate coagulopathy by influencing platelet function, further complicating patient outcomes [24].

Despite being a recommended treatment in severe bleeding [19], TXA was administered to only 28% of patients. TXA is generally not recommended to patients with GI-bleeding [25]. In this study we observed that 24% of these patients received TXA. Among patients with bleeding related to other causes only 32% got TXA. This low rate suggests potential barriers to its use, possibly due to prioritization of other life-saving interventions in hemodynamically unstable patients.

The mean time from ambulance arrival at the scene to hospital admission was 53 min, with transport times reaching up to 180 min in rural areas. Given the high prevalence of coagulopathy and a high SI in combination with prolonged transport times, early administration of dried plasma might be beneficial [13]. Dried plasma offers logistical advantages over fresh blood products, which require a cold chain and are not widely available in ambulances.

This observational study has limitations, including variations in patient inclusion and resuscitation protocols across different ambulance providers. Estimating blood loss and fluid administration remains challenging in prehospital settings. Also, patients with incomplete datasets were included, which led to missing data in some variables. Additionally, the study did not account for pre-existing anticoagulant use due to underlying medical conditions, which may have influenced the high coagulopathy rate.

These findings emphasize the need for improved prehospital management of bleeding patients. Blood loss over 500 mL and a Shock Index above 1.3 were key predictors of poor outcomes, highlighting the potential benefit of earlier blood product administration. Optimizing TXA use and exploring dried plasma for prehospital care might reduce coagulopathy and enhance patient outcomes, particularly in cases with long transport times.

## Conclusion

A high proportion of the bleeding patients treated with crystalloid fluid resuscitation in the regular ambulance service have a coagulopathy and a high shock index upon arrival at the hospital. A high shock index predicted the need for blood transfusions, and a prehospital bleeding of more than 500 mL predicted mortality. Gastrointestinal bleeding was the major cause of severe bleeding, with trauma as the second most common. Prehospital use of blood products is increasingly requested where dried plasma may be an alternative in the regular ambulance service. The possible advantage needs however to be further investigated.

## Data Availability

No datasets were generated or analysed during the current study.

## Abbreviations

aPTT: Activated partial thromboplastin time
CRF: Case report form
NACA: National advisory committee for aeronautics
PT-INR: Prothrombin time/ international normalised ratio
RBC: Red blood cells
SBT: Systolic blood pressure
SI: Shock index
TXA: Tranexamic acid
